# Aesthetics by Numbers: Links between Perceived Texture Qualities and Computed Visual Texture Properties

**DOI:** 10.3389/fnhum.2016.00343

**Published:** 2016-07-21

**Authors:** Richard H. A. H. Jacobs, Koen V. Haak, Stefan Thumfart, Remco Renken, Brian Henson, Frans W. Cornelissen

**Affiliations:** ^1^Laboratory for Experimental Ophthalmology, University Medical Center Groningen, University of GroningenGroningen, Netherlands; ^2^Donders Institute for Brain, Cognition and Behavior, Donders Center for Cognition, Radboud UniversityNijmegen, Netherlands; ^3^Donders Institute for Brain, Cognition and Behavior, Centre for Cognitive Neuroimaging, Radboud UniversityNijmegen, Netherlands; ^4^Profactor GmbHSteyr-Gleink, Austria; ^5^Research Unit for Medical-Informatics, RISC Software GmbH, Johannes Kepler University LinzLinz, Austria; ^6^BCN NeuroImaging Center, School for Behavioral and Cognitive Neurosciences, University Medical Center GroningenUniversity of Groningen, Groningen, Netherlands; ^7^School of Mechanical Engineering, University of LeedsLeeds, UK

**Keywords:** aesthetics, texture perception, semantic differential, features, evaluative, descriptive, beauty, roughness

## Abstract

Our world is filled with texture. For the human visual system, this is an important source of information for assessing environmental and material properties. Indeed—and presumably for this reason—the human visual system has regions dedicated to processing textures. Despite their abundance and apparent relevance, only recently the relationships between texture features and high-level judgments have captured the interest of mainstream science, despite long-standing indications for such relationships. In this study, we explore such relationships, as these might be used to predict perceived texture qualities. This is relevant, not only from a psychological/neuroscience perspective, but also for more applied fields such as design, architecture, and the visual arts. In two separate experiments, observers judged various qualities of visual textures such as beauty, roughness, naturalness, elegance, and complexity. Based on factor analysis, we find that in both experiments, ~75% of the variability in the judgments could be explained by a two-dimensional space, with axes that are closely aligned to the beauty and roughness judgments. That a two-dimensional judgment space suffices to capture most of the variability in the perceived texture qualities suggests that observers use a relatively limited set of internal scales on which to base various judgments, including aesthetic ones. Finally, for both of these judgments, we determined the relationship with a large number of texture features computed for each of the texture stimuli. We find that the presence of lower spatial frequencies, oblique orientations, higher intensity variation, higher saturation, and redness correlates with higher beauty ratings. Features that captured image intensity and uniformity correlated with roughness ratings. Therefore, a number of computational texture features are predictive of these judgments. This suggests that perceived texture qualities—including the aesthetic appreciation—are sufficiently universal to be predicted—with reasonable accuracy—based on the computed feature content of the textures.

## Introduction

Can aesthetic appreciation of textures be predicted based on computed visual features? That is the question addressed in the present work that arose from the Syntex project (http://visualneuroscience.nl/syntex). Aesthetics often refers to beauty and related judgments, such as preferences. In a broader sense, it often refers to other impressions, such as the judgment of naturalness. Both interpretations apply to this article; its focus is on beauty, but we also consider other judgments about visual textures.

We use the following working definition of texture: any pattern in which no single object outline can be discerned. We used “single” because an outline of one stone would count as an object, but a field of stones would count as a texture. Textures typically contain repetitive information. For the present study, color was defined as an integral part of textures or surface properties.

Visual and tactile textures are widely used in industrial design, art, and architecture to convey information (e.g., about the atmosphere or safety of buildings, or the strength, quality, or intended use of objects) and to influence aesthetic experience. Despite this widespread use, until recently there have been relatively few systematic attempts to reveal systematic relationships between such perceived aesthetic qualities and the texture’s computed visual features. The Syntex project and its derivatives also addressed the impact of visual textures on aesthetic experiences in a number of previous publications (Thumfart et al., 2008, 2011; Liu et al., 2015).

### Using Textures to Examine Aesthetic Responses

The study of texture processing is interesting in itself because evidence is accumulating that textures are processed in dedicated visual processing regions, which are located mainly along the medial visual cortex (Puce et al., 1996; Peuskens et al., 2004; Cant and Goodale, 2007; Hiramatsu et al., 2011; Jacobs et al., 2014). We consider textures or surfaces as the complement of shapes or outlines. Texture information can be quantified as the degree to which a feature is present. For outline stimuli, in which texture information is dropped, only things such as the length of outlines, the position of certain elements, or the number of elements can be quantified, along with features such as contrast which can also be computed for textures. When using natural stimuli such as faces, texture information can be quantified for the entire picture, but this would disregard differences in various parts of the picture; e.g., the frequency content of a face would differ from the frequency content of the hair or of the background of the face, resulting in average values which reflect neither. We consider these to be issues that can potentially affect any human output (judgments or physiological responses). Studying texture perception may therefore lead to insights into human perception that may not be found when using other stimulus types. In addition, textures provide important clues about material properties. Understanding texture perception will therefore contribute to our understanding of the perception of material properties.

Texture processing is not only inherently interesting. If we improve our understanding of texture processing mechanisms, this may shed light on the processing of other stimuli that are more typically investigated in aesthetics research, such as photographs of faces or objects or various categories of painting (which also contain texture). Moreover, the results could possibly point out confounds in other studies. Compared to such relatively complex stimuli, the use of textures has advantages, such as minimizing semantic associations that are hard to control for. Semantic information has been shown to be an important factor for determining preferences (Berlyne, 1970; Martindale et al., 1990). With textures, semantic influences are attenuated, although some textures may still elicit associations through the recognition of the materials of which they are composed (e.g., stone, wood, silk or fur). A final advantage of using textures over more complex stimuli is the availability of a large number of algorithms to compute image features, allowing quantification of their relationship to perceived texture qualities. For this reason, we refer to our approach as: aesthetics by numbers.

### Previous Research into Texture Perception

In the visual domain, studies examining texture perception have primarily focused on lower-level texture processing such as texture segmentation and discrimination (Julesz, 1981; Bergen and Adelson, 1988; Knill et al., 1990; Landy and Bergen, 1991; Williams and Julesz, 1992; Victor and Conte, 1996; Merigan, 2000; Sireteanu et al., 2005; Victor et al., 2005; Ben-Shahar, 2006; Abbey and Eckstein, 2007; Yeshurun et al., 2008; Hollingworth and Franconeri, 2009). Studies of higher-level processing of visual textures have focused on judgments of appearance and material properties related to glossiness (Pont and te Pas, 2006; Motoyoshi et al., 2007a), illumination (Pont and te Pas, 2006), metallic appearance (Motoyoshi et al., 2007b), transparency (Watanabe and Cavanagh, 1993; Fleming and Bülthoff, 2005), estimated weight (Buckingham et al., 2009), roughness (Ho et al., 2006), slipperiness (Lesch et al., 2008), complexity and self-similarity and liking (Bies et al., 2016; Güçlütürk et al., 2016), and the relationship between perceived material properties and material categories (Fleming et al., 2013).

The number of studies investigating preferences for textures or features that can be considered texture features (e.g., Soen et al., 1987; Aks and Sprott, 1996; Schira, 2003; Fleming et al., 2013) is greatly exceeded by the vast number of studies devoted to understanding the affective responses to objects. Aesthetics research has often focused on stimuli such as paintings or faces for which feature information is hard to control—let alone that this has even been attempted. Several studies have found relationships between preference and color features (Ball, 1965; Valdez and Mehrabian, 1994). Some studies have examined the frequency content and self-similarity of paintings, with or without relating this aspect to actual beauty judgments (Redies et al., 2007; Graham and Redies, 2010; Mallon et al., 2014). In the Syntex-project and the current article we specifically sought to address the relationship between texture features and aesthetics.

### Evidence for a Textural Influence on Preferences

The recency of the interest in the relationships between textural image features and beauty ratings is somewhat surprising, given the many long-standing indications in the literature that texture may have an impact on preference. Such indications come from studies investigating the relationship between preference and fractal dimension (Aks and Sprott, 1996; Spehar et al., 2003; Juricevic et al., 2010; Spehar and Taylor, 2013), entropy (Stamps, 2002), spatial frequency content (Soen et al., 1987; Kawamoto and Soen, 1993; Schira, 2003) or certain colors (Valdez and Mehrabian, 1994; Jacobs et al., 2010) of stimuli (usually not textures—but such features are also present in textures). Also work showing that paintings contain certain spatial frequency characteristics (Redies et al., 2007; Graham and Redies, 2010) is suggestive of such preference. Moreover, texture strongly influences facial attractiveness (Jones et al., 2004). In line with the reported relationship between spatial frequencies and beauty ratings, the brain responses to affective stimuli—such as expressive faces—depend on the frequency bands present in the stimulus (Vuilleumier et al., 2003; Holmes et al., 2005; Alorda et al., 2007; Delplanque et al., 2007). Moreover, brain centers regarded as emotion processors (such as the amygdala) respond to features such as angularity (Bar and Neta, 2007), which are both object and texture features.

To summarize the above, there are indications that texture features influence beauty ratings. Until recently, these influences have not yet been systematically investigated. To do so, we decided to perform two exploratory experiments and a computational analysis to establish the degree to which computed visual texture features influence beauty and other high-level judgments.

Our present study differs in a number of ways from some of the earlier work from the Syntex-consortium (Thumfart et al., 2008, 2011; Liu et al., 2015). First, we opted for a semantic differential approach in which—based on a factor analysis of a larger number of judgments—we select a small number of judgments that best represent the observers’ judgment space, rather than* a priori* assigning judgments to different “cognitive layers” (Thumfart et al., 2008, 2011; Liu et al., 2015). Second, we also used factor analysis for selecting the relevant computational features (rather than the Laplacian Score employed by Liu et al., 2015). Third, we emphasize the relevance of single features to the selected judgments, rather than the overall performance of a model, as Liu et al. did.

To give an overview of the present study, we first conducted an experiment for selecting the appropriate textures to use, and one to select appropriate adjectives for use in the judgments. Next, we conducted two separate semantic differential experiments, in which we focus on revealing the relationships between various judgments on textures and selecting the most representative ones (based on factor analysis). Finally, in a computational analysis, we address the relationships between computed texture features and the selected judgments.

## Experiment 1: Texture Selection

The aim of this experiment was to select the textures for use in semantic differential experiment 1 (reported below).

### Methods

#### Participants

Twenty four participants (12 males, age range 18–29 years) participated. All participants had normal or corrected to normal vision. Our entire study conformed to the tenets of the Declaration of Helsinki. The experiments were carried out as part of a psychology bachelor’s course. The ethical review board of the Department of Psychology of the University of Groningen approved the study. Participants gave their written informed consent prior to participation. All participants were students in higher education and received course credits for their participation.

#### Equipment and Software

Experiments were run on a MacBook pro under Mac OS X (Apple, Cupertino, CA, USA), using Matlab (Mathworks, Natick, MA, USA) with the Psychophysics toolbox extensions (Brainard, 1997). Stimuli were presented on a 30” Apple Cinema HD Display monitor.

#### Stimuli

**Figure 1:**
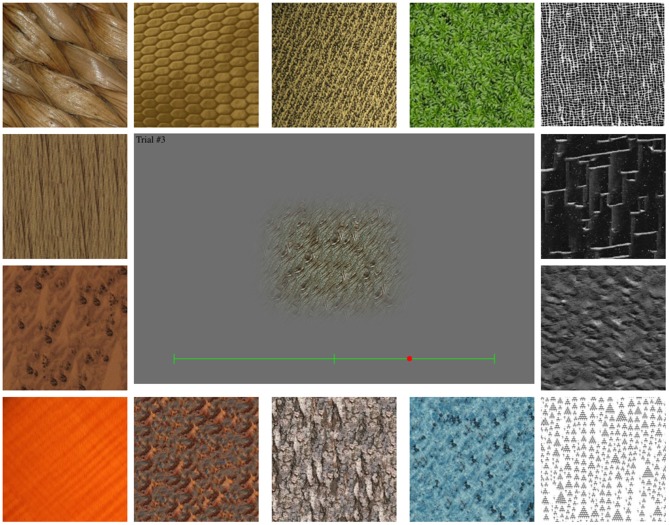
**Example textures.** Thumbnails of textures used in the experiments. The enlargement shows a texture sample, as displayed on screen, with a green slider bar at the bottom.

From a large database (available on request; see Figure 1 for examples), taken from various sources, a pre-selection of 300 textures was made, based on criteria such as the absence of object outlines, and the elimination of very similar textures. The visual angle of the textures ranged from 3.3 to 32° in height, and from 5.7 to 37° in width.

#### Procedure

Textures were presented on the screen one by one. Participants indicated their preference by adjusting the position of a slider at the bottom of the screen (Figure 1) by moving a mouse along a bar corresponding to the judged dimension. They indicated their judgment by clicking on the desired location (right = beautiful; left = ugly/not beautiful).

Satisfaction with the judgment was indicated by pressing a mouse button, after which the screen went blank. The next trial started 1000 ms later. Instructions were given orally, as well as written on the screen, prior to the start of each run. All textures were presented once in a block, in random order. Participants were asked to use the entire range of the slider, and to not necessarily regard the central point as being “neutral”. In order to give a sense of the range of stimuli they would see, and to practice the procedure, a few test trials were performed by the participant before the actual start of the experiment. Participants were asked to respond based on their first impression. Participants performed the test individually. The experiment was performed in a room that was dark, except for the illumination provided by the screen.

#### Analysis and Selection

Based on the average rank order over participants, the 20 most and 20 least liked textures were selected, as well as 20 from the middle of the range. Note: we determined that such a selection of the most extreme textures is necessary to obtain reliable beauty ratings (see Supplementary Material, part I). We consider that this is likely to also enhance our ability to discover relationships between specific features and judgments.

### Results

**Figure 2:**
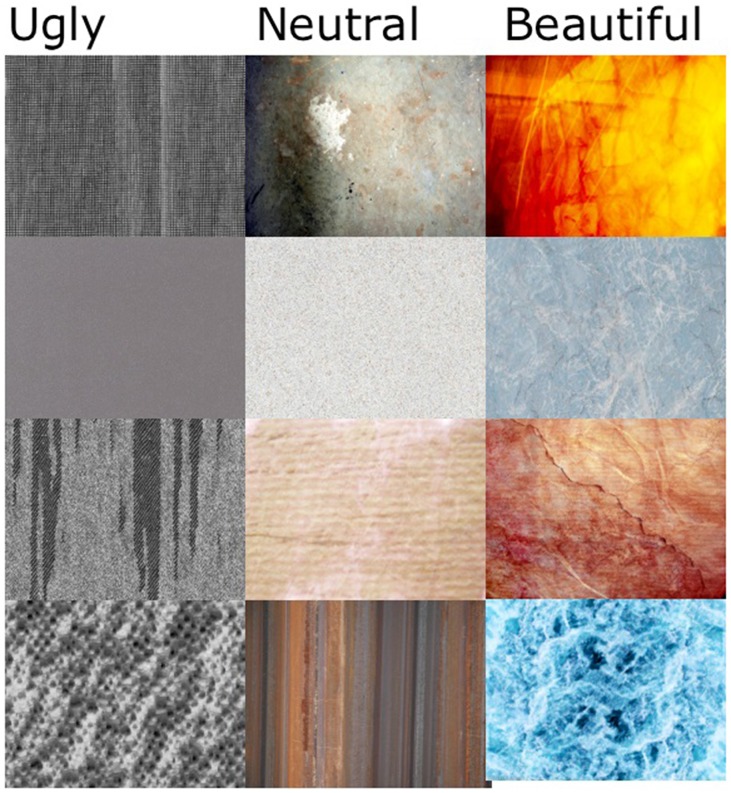
**Examples of textures rated high, low, and average on beauty.** Twenty of each were selected for use in the first semantic differential experiment.

The texture selection experiment yielded a rank ordering of the 20 most and the 20 least liked textures, as well as 20 from the middle of the range. Four of each category are displayed in Figure 2.

## Experiment 2: Adjective Selection

The aim of this experiment was to select the adjectives for use in the first semantic differential experiment (reported below).

### Methods

#### Participants

Seventeen participants (9 males, age range 18–28 years), different from those who participated in experiment 1, took part. All participants had normal or corrected to normal vision.

#### Stimuli, Equipment and Software

Stimuli were the textures selected in the experiment 1. Equipment and software was identical to that used in experiment 1. This experiment was performed in a dimly lit room, to allow participants to write down their responses.

#### Procedure

This experiment consisted of a “adjective association” and a “triad” task. It was performed in groups of up to five participants, who were seated 2–3 meters from the screen. Participants noted their responses on paper, and the experiment leader paced the task according to the speed of the group. First, in the adjective association task, participants viewed the textures, and wrote down any words (up to five) occurring to them. In the triad task, the textures (halved in size) were presented in groups of three. Participants’ task was to pick the one that did not belong to the others, and write down why it was different (the “Minimum Context Card Form” of construct elicitation by triads of elements (Kelly, 1955; Fransella and Bannister, 1977)). Both in the adjective generation task and in the triad task each texture was presented only once.

#### Analysis

Similar adjectives were grouped together. Grouping was according to a consensus judgment between four experimenters. A count was made of the occurrence of adjectives in each group, to arrive at the adjectives that were most frequently used.

### Results

Participants generated many different adjectives, which could be grouped into categories without much disagreement between experimenters. The adjectives generated contained not only many descriptive adjectives, like rough and hard, but also many subjective adjectives, such as boring, artistic, beautiful, warm, and cheerful. The adjective generation experiment yielded a list of adjectives categorized in groups (Table 1). For the most frequently mentioned groups, one representative adjective was chosen for use in the first semantic differential experiment. Words relating to contrast and luminance were excluded, because we expected they would relate too obviously to their computed counterparts.

**Table 1:** **Words generated in the adjective generation experiment, grouped together according to similarity**.

Generated adjectives		
Adjectives	Adjectives	Word count
*Ugly*	*Beautiful, gorgeous*	63
*Smooth*, flat, slender	*rough*, hefty, granular	82
*Cold*, chilly, cool,	*warm*, sunny, cheerful,	172
not sunny	summertime, happy
*soft*, not hard	hard	40
Dark, unlit, night	light, bright	318
boring	Exciting, snazzy,	22
	snappy, touching,
	thrilling, *interesting*
not artistic	artistic, picturesque,	7
	skilful
not much color, black-white,	*colorful*, fierce	235
faint, *colorless*	colors, color shades
*old*, outdated, antique	*young*, new	47
*fuzzy*, unclear, undefinable	not vague, *sharp*, clear	179
artificial	natural	78
irregular	regular	8
little contrast	a lot of contrast	37

*The extremes of the relevant dimension are arranged in different columns, with a third column mentioning the number of times these were mentioned. Words selected for use in the first semantic differential experiment are italicized*.

## Semantic Differential Experiment 1

We performed a semantic differential experiment using the textures and judgments obtained through the two selection experiments reported above. Our aim was to examine the relationships between the different judgments, and to determine the dimensionality of the judgment space.

### Methods

#### Participants

There were 19 participants (12 male; age range 18–29 years). This was a subgroup of the participants that had also participated in selection experiments 1 or 2.

#### Equipment and Software

The equipment and software was the same as that used in experiment 1. The experiment was performed in a room that was dark except for the illumination provided by the screen.

#### Stimuli

Sixty texture stimuli (see Figures 1, 2 for examples)—selected in experiment 1—were used. Stimuli were displayed on a gray background, into which they were blended smoothly (see Figure 1). Viewing distance was about 70–80 cm.

#### Procedure

In a trial, participants were presented with a texture on a computer screen, and were asked to judge it on one of several dimensions. Judgments were made for the following dimensions: beautiful-ugly, smooth-rough, hard-soft, colorfulness, warm-cold, young-old (age), natural-artificial, fuzziness-sharpness, and interestingness-boringness. These dimensions were selected based on the results of experiment 2. As in experiment 1, participants indicated their preference by adjusting the position of a slider at the bottom of the screen. The poles of the judgments were randomly assigned to either the left or right side of the slider bar. For each observer, dimensions were evaluated in separate runs, the order of which was randomized.

#### Analysis

The individual judgments, per adjective, were linearly re-scaled to a range between –100 and +100. Next, the average judgments were computed over subjects. The resulting scores were subjected to a factor analysis, using Varimax rotation and Kaiser normalization (Kaiser, 1958) in SPSS. The number of factors to be retained was determined by parallel analysis (Kaufman and Dunlap, 2000; Hayton et al., 2004), using 100 permutations of the average judgments about the textures. The eigenvalues exceeding those for the permuted data were taken as indicating factors to be retained.

### Results

**Figure 3:**
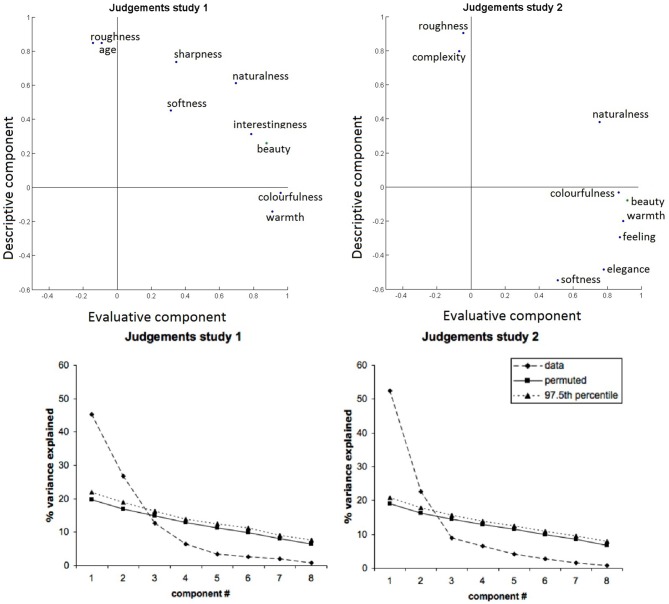
**Judgments loadings on the varimax rotated factors, for both studies.** Two factors were retained. Most judgments load selectively on one of both factors. Results for the first experiment (left panel) and second experiment (right panel) are very comparable.

For the judgments, parallel analysis indicated that two factors should be retained in a factor analysis (Figure 3, bottom left panel). The first two factors explained 73% of the variance in the judgments.

Results (Figure 3, top left panel) show that colorfulness, warmth, beauty and interestingness load strongly on the first factor, while roughness and age load strongly on the second factor. Naturalness fell in-between, and the hard-soft dimension had low loadings on both components. Results for a three-component solution are shown in the Supplementary Material, (part III).

### Conclusion

We found that two components could adequately capture most of the variation (73%) in the judgment space. This indicates that many judgments were highly related. Various authors (e.g., Mandler and Shebo, 1983; Jacobsen et al., 2006) have distinguished between evaluative and descriptive judgments, and we find a similar distinction for the texture stimuli. The first component received high and exclusive loadings of judgments which can be qualified as evaluative in nature: beauty, warmth, colorfulness, and interestingness. Intuitively, it makes sense that textures judged to be beautiful tend to also be judged positive in other respects, such as elegance and warmth. These aesthetic judgments seem to be based on perceived colorfulness to a large extent. The second component received high and exclusive loadings of more descriptive judgments, namely roughness and age.

## Semantic Differential Experiment 2

A second semantic differential experiment was undertaken to verify the results of the first one, yet using somewhat different judgments and texture stimuli. We also did this because replications are required for small sample sizes (Guadagnoli and Velicer, 1988). In this way, we hoped to verify the robustness of the results. While the selection of texture stimuli based on beauty ratings (as in experiment 1) may have enhanced our ability to find relationships with beauty, it may also have biased results in undesirable ways. Therefore, here we opted not to do so again. Rather, we chose different texture stimuli from our set by including textures covering much of the feature space. Textures were selected by eye since, due to the large number of features compared to the relatively limited number of texture stimuli, it proved impossible to do this systematically and rigorously. Another difference with the first experiment is that we equalized the size of the different stimuli, as is common practice in psychological experiments. We also chose partially different judgments, while sticking to others, to strike a balance between replicating the previous results and generalizing our results to new judgments (output) and textures (input). Despite the somewhat different set of judgments and textures, we anticipated that results from this second experiment would agree with those of the first experiment.

## Materials and Methods

### Participants

There were 20 participants (10 males; age range 20–29 years), none of whom had participated in the first semantic differential experiment nor in selection experiments 1 or 2.

### Equipment and Software

Equipment and software was largely as described in semantic differential experiment 1, except for the display used, which was a 19” LaCie CRT monitor.

### Stimuli

**Figure 4:**
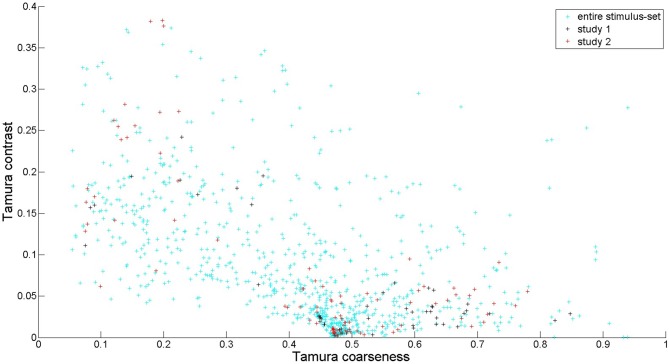
**Feature values of textures for Tamura coarseness and contrast.** All textures in our database are depicted in cyan (light blue). The ones used in semantic differential experiment 1 are depicted in black, and the ones used in semantic differential experiment 2 are depicted in red. Tamura coarseness was already well covered in experiment 1. In experiment 2, some higher values for Tamura contrast were included to have a better coverage of this feature. We could not take into account all possible combinations of features, and in this case one can see that the combination of high Tamura coarseness and high Tamura contrast was not included.

Seventy texture stimuli were taken from the database containing textures originating from various sources. All textures were re-sized to 24.2 (width) by 20.2 (height) degrees, by cropping larger textures, or by “growing” smaller textures, using a standard algorithm (Efros and Leung, 1999). Stimuli were displayed on a gray background, into which they blended smoothly (see Figure 1). Viewing distance was about 70–80 cm. Textures were selected to cover the range of feature values better than in semantic differential experiment 1, as illustrated in Figure 4.

### Procedure

The procedure was largely identical to the procedure for semantic differential experiment 1, with the following exceptions: judgments were made for the dimensions beautiful-ugly, smooth-rough, hard-soft, colorfulness, warm-cold, complex-simple, natural-artificial, elegance, and feeling (“How does this texture make you feel?”: positive-negative).

### Analysis

The analysis was as described for semantic differential experiment 1.

### Results

Results are displayed in the right panels of Figure 3. Parallel analysis indicated that two factors should be retained in a factor analysis (bottom right). The first two factors together explained 76% of the variance in the judgments. The first factor received high loadings of the judgments colorfulness, beauty, warmth, feeling, and elegance, while the second factor received high loadings of roughness and complexity.

### Conclusion

Results of both studies were highly similar, despite the involvement of only a limited number of participants, the use of different textures, and the substitution of some judgments by others. In both studies, parallel analysis indicated that two components sufficed to capture most of the variability in the judgments. Also in both studies, beauty, colorfulness, and warmth load highly and exclusively on the first component, while roughness loads highly and exclusively on the second component. Naturalness falls in-between these other judgments, with a moderately high loading on both components. The hard-soft dimension had relatively low loadings on both components, and did not seem to be captured well by the two-component solution. The robustness of the results is indicated by the judgments which were included in both studies. As in semantic differential experiment 1, the results support our labeling of the first component as evaluative in nature and the second component as descriptive in nature. Based on the results of both semantic differential experiments, beauty appears a good pick for a judgment representing the evaluative component (and has an obvious relevance in an aesthetic study), while roughness appears to well represent the descriptive component.

## Computational Analysis: The Relationship Between Features and Judgments

The goal of this analysis is to establish the degree to which computed visual texture features correlate with the beauty and roughness judgments (being chosen as representative judgments for the two axes in judgment space uncovered in the two semantic differential experiments). We explore such relationships, as these might be used to predict perceived texture qualities.

### Methods

#### Feature Computation

A more elaborate description of our texture features and their computation is provided in the Supplementary Material (part II). Here we provide a brief overview.

Computed features are based on Gray-Level Co-occurrence Matrices, a set of features related to psychological judgments (Tamura features), Neighborhood Gray-Tone Difference Matrices, the Fourier spectrum, Gabor energy features, and features expressing the presence of colors, brightness, and saturation.

The Tamura features are based on psychological evaluations, and comprise coarseness, contrast, directionality, line-likeness, regularity, and roughness. The Gray Level Co-occurrence Matrices are used to compute statistical properties like entropy, energy, and homogeneity and indicate how often particular gray levels co-occur at a certain distance. For our purposes, we computed them for distances of 1, 2, 4, and 8 pixels. A Neighborhood Gray Tone Difference Matrix is a vector containing, for each gray level, a sum of the differences in gray tone with all the surrounding pixels, for each pixel with that gray tone. The size of the neighborhood is variable, and we computed matrices for sizes of 3 by 3 and 5 by 5 pixels. Based on these matrices, the features coarseness, contrast, busyness, complexity, and strength were computed.

Fourier features are based on the spatial frequencies in the brightness variations. The extent to which a certain spatial frequency is present is expressed as its energy or power. First, a two-dimensional image is transformed into the frequency domain using the fast Fourier transform, to obtain the Fourier spectrum. Each component of the spectrum is represented by a complex number that describes a frequency in the two-dimensional image by means of amplitude and phase. The component coordinates in the spectrum determine the frequencies wave length and direction. The spatial frequency with highest wave length (uniform signal, i.e., average brightness) is represented in the center of the spectrum, while high frequencies can be found on the outside.

The average energy of circular bands around the average brightness was computed for different radii. Also, the energy of wedges with their peak at the average brightness was computed, yielding a measurement of the orientation of the image. In this way, 12 circular energy features and 24 wedge energy features were computed, each reflecting the presence of information at a different spatial frequency (circular rings) and at a different orientation (wedges). In addition, a number of features summarizing their distribution were computed. Similar to Fourier features, Gabor features capture the spatial frequencies in pictures, but they preserve some spatial information. The human visual system is known to contain cells that work as Gabor filters. Gabor “energy”, over the entire texture, was computed for four spatial frequencies, in six orientations. Average saturation and intensity were assessed after converting the image from RGB to HSV color space. The presence of the colors red, green, yellow, cyan, blue, and magenta, was computed by partitioning the hue component of HSV color space into six sectors, and counting the relative frequency of pixels within each sector. The sector frequency was normalized to the average image value and saturation.

We verified that the use of the texture growth algorithm did not affect the feature values by computing the texture features before and after application of the algorithm. The feature values after application of the algorithm were obtained by averaging the feature values over 30 patches of each texture. We determined the percentage of feature values falling outside a range of two standard deviations (the 95% confidence interval) from the original textures, in the distribution of original texture feature values. Across all features and textures, 0.7% of the feature values fell outside this interval, indicating that the feature values were hardly affected by the texture growth algorithm.

### Results

#### Linear Regression Between Judgments and Feature Factors

Factor analysis was performed with the 20 feature factors explaining most of the variance in the feature values. Although the parallel analysis indicated that 10 factors should be retained, we doubled the amount because we did not want to miss any potentially relevant factors. To some extent this—admittedly non-rigorous—decision is justified by the fact that a parallel analysis only evaluates the variance in the computational feature values. Therefore, this does not guarantee relevance of the computed factors—or the retained computational features—for the beauty or other judgments. In particular, one factor containing color information would have been missed if we had retained only 10 factors, and color appears relevant in the light of the colorfulness judgment relating to beauty (see above) and of previous results indicating that people fixate more on patches containing color information when judging for beauty (as compared to roughness; Jacobs et al., 2010). Subsequently, a linear regression was performed between these 20 feature factors on the one hand, and the beauty and roughness judgments on the other, in order to determine which feature factors significantly correlated with the beauty and roughness judgments.

The 20 feature factors together explained 63% of the variance in the feature values. They explained 59% of the variance in the beauty ratings and 50% of the variance in the roughness ratings.

Feature factors 4, 6, 9, 13, and 17 exerted a significant effect on beauty judgments, and Feature factors 3 and 9 exerted a significant effect on roughness (Table 2). The features loading strongest, whether positively or negatively, on these factors are displayed in Table 2. Based on these features, we labeled Feature Factor 4 as an intensity variation factor, Factor 6 as a low spatial frequency Gabor factor, Factor 9 as an intensity factor, Factor 13 as a saturated redness factor, Factor 17 as a directionality (diagonality) factor, and Factor 3 as a uniformity factor.

**Table 2:** **The feature factors that significantly correlated with beauty and roughness ratings and their constituent features**.

Judgment	Factor	Features	*r*	Description
Beauty	4 *r*^2^ = 0.22 intensity variation	Mean correlation (*d* = 8) C_energy2 Roughness Mean correlation (*d* = 4) C_energy3	0.39 0.40 0.43 0.44 0.33	Mean intensity variation of pixel pairs at a distance of 8 pixels Presence of low spatial frequency content in the texture Tamura roughness, high if both coarseness and contrast are high Mean intensity variation of pixel pairs at a distance of 4 pixels Presence of low spatial frequency content in the texture

	6 *r*^2^ = 0.09 Gabor frequency	O2s1 O3s1 O6s1 O5s1 C_energy5	0.11 0.11 0.14 0.15 0.03	Low spatial frequencies from the Gabor domain, with different orientations Presence of low spatial frequency (Fourier) content in the texture

	9 *r*^2^ = 0.04 intensity	Avg. texture int. (*d* = 8) Avg. texture int. (*d* = 4) Avg. texture int. (*d* = 2) Avg. texture int. (*d* = 1) Average intensity	0.18 0.18 0.19 0.20 0.33	Mean over the average texture intensity; i.e., average texture intensity
	13 *r*^2^ = 0.21 saturation, intensity	Average saturation Degree of redness Regularity Average intensity R_sumVar	0.45 0.26 −0.07 0.33 −0.39	Range of the weighted sum of the GLCM secondary diagonal entries

	17 *r*^2^ = 0.08 directionality	Wedge energy 67.5 Wedge energy 60 Wedge kurtosis Wedge energy 52.5 Wedge energy 70.5	0.26 0.23 0.00 0.11 0.20	Presence of texture elements with orientation 67.5° from vertical Presence of texture elements with orientation 60° from vertical Skewness of the distribution of wedge energy features Presence of texture elements with orientation 52.5° from vertical Presence of texture elements with orientation 70.5° from vertical

Roughness	3 *r*^2^ = 0.34 uniformity	Mean angular second moment (*d* = 2) Mean angular second moment (*d* = 1) Mean angular second moment (*d* = 4) Mean angular second moment (*d* = 8) Mean entropy (*d* = 2)	−0.44 −0.43 −0.45 −0.45 0.58	Mean over the sum of squared GLCM entries. A measure of uniformity Mean entropy

	9 *r*^2^ = 0.13 intensity	Avg. texture int. (*d* = 8) Avg. texture int. (*d* = 4) Avg. texture int. (*d* = 2) Avg. texture int. (*d* = 1) Average intensity	−0.39 −0.40 −0.40 −0.39 −0.47	Mean over the average texture intensity; i.e., average texture intensity

*The first column lists the judgments. The second column lists the number of the feature factor, and a label attached to these factors, based on the most relevant features. The third column lists the features with the highest absolute loadings on each of the relevant feature factors. The fourth column lists the correlation coefficients per feature, to confirm the relevance of these features, and to ascertain the directionality of the relationship to the ratings. The fifth column provides a brief description of the features*.

Factors exerting a significant effect on beauty, at a Bonferroni-corrected two-sided α-threshold of 0.05/20 = 0.0025 (109 degrees of freedom per test), were Factors 4 (*t* = −6.84, *p* < 0.0001), 6 (*t* = −4.03, *p* = 0.0001), 9 (*t* = 3.14, *p* = 0.0022), 13 (*t* = −5.38, *p* < 0.0001), and 17 (*t* = −3.17, *p* = 0.0020). Factors exerting a significant effect on roughness were Factors 3 (*t* = −6.90, *p* < 0.0001) and 9 (*t* = −3.54, *p* = 0.0006).

#### The Direction of the Relationships

To determine the direction of the relationships, and to confirm the findings of our analysis, direct correlations were computed between beauty and the features loading strongly on the feature components that were relevant for beauty. Similarly, correlations were computed between roughness and the features loading strongly on the feature components that were relevant for roughness.

Low spatial frequencies, computed according to Fourier principles (as in Factor 4), were associated with higher beauty ratings. Accordingly, high spatial frequencies computed according to Gabor principles (as in Factor 6) were negatively associated with beauty ratings. Saturation and redness were positively associated with beauty ratings. The presence of diagonal elements in the textures (the wedge energy features in Factor 17) was associated with higher beauty ratings. Uniform textures were rated as smooth or not rough. High average intensity (luminance) led to low roughness ratings.

#### Conclusion

A number of feature factors, and through these a number of computational features, correlated significantly with beauty and roughness ratings. In other words, both beauty and roughness judgments about textures show systematic relationships to computed visual features present in the textures.

## General Discussion

Even though individuals may differ in their aesthetic judgments, in this study we have shown that there is a common element in their judgments which can be related to specific computed visual features present in the textures. Below, we discuss this finding as well as our other results in more detail.

### A Two-Dimensional Judgment Space Suffices to Capture Most Variability in Perceived Texture Qualities

We found that two judgment factors captured 73% (experiment 1) and 76% (experiment 2) of the variance in the judgments. The first factor was associated with the judgments of beauty, elegance, “feeling”, warmth, colorfulness, and interestingness. With the exception of colorfulness, these judgments seem to have an affective or evaluative element in common. The second factor had high and exclusive loadings of roughness, complexity, and age, and seems to be more descriptive in nature. These two factors are orthogonal, meaning that the judgments loading exclusively on one component are unrelated to the judgments loading exclusively on the other component. On the other hand, the judgments loading high on the same factor are highly correlated. The two factors together make up a two-dimensional judgment space that captures most of the variability in the perceived texture qualities. This finding suggests that observers use a relatively limited set of internal scales on which to base various judgments, including aesthetic ones.

Rao and Lohse (1996) used a different technique to identify relevant dimensions for texture perception: subjects grouped textures into categories of their own choice. The data were analyzed using multidimensional scaling and hierarchical cluster analysis. Based on this, they identified the dimensions of repetitiveness-irregularity, directionality, and simplicity-complexity as important for texture perception. It is striking that: (1) except for regularity (mentioned eight times) which seems highly similar to repetitiveness, our participants did not spontaneously generate words pertaining to these dimensions; and (2) our semantic differential studies did not distinguish between these dimensions. At the same time, it is striking that Rao and Lohse (1996) did not identify components relating to more subjective aspects, such as beauty, warmth, and naturalness. What may underlie the differences between the adjectives we obtained, and the dimensions obtained by Rao and Lohse (1996)? A first possibility is the difference in methodologies. We had subjects generate words, while Rao and Lohse (1996) had subjects classify textures, without an explicit reference to words or adjectives. Possibly, observers use more “objective” (in the sense of higher inter-subject agreement) criteria for categorizing than for labeling textures. A second possibility lies in the different stimulus sets used: Rao and Lohse (1996) used 30 textures from the Brodatz album, which are limited in that they are all gray-scale pictures, and most of the repetitive textures have horizontal and/or vertical orientation. Color or colorfulness might be an important factor eliciting more subjective qualifications, like beauty, as might be inferred from our semantic differential studies, where both beauty and colorfulness judgments load highly on the same component. We used a much broader set of textures, which included Brodatz textures, but also a lot of other textures, obtained from commercial or other websites. These different texture sets may have elicited different associations.

These two judgment factors that we find correspond well to judgment factors obtained in other studies, where different stimuli (i.e., non-textures) were employed (Osgood et al., 1957; Takahashi, 1995). Such studies typically found an evaluative factor with high loadings of subjective judgments such as beauty, elegance, warmth, and another factor with high loadings of more descriptive judgments such as roughness. These studies labeled these factors as “evaluative” and “potency”, respectively. We labeled the second factor as descriptive, following Jacobsen and Höfel (2003).

### Computed Visual Texture Features are Predictive of Perceived Texture Qualities

We adopted an exploratory approach, and used a diverse set of textures, features, and judgments, to find relationships between texture features and judgments, without restricting ourselves to features or judgments reported in the literature. Because so many parameters lead to a loss of degrees of freedom in statistical testing, we reduced feature space to the 20 most important factors. The 20 feature factors together explained 63% of the variance in the feature values. They explained 59% of the variance in beauty judgments, and 50% of the variance in roughness judgments. We identified five feature factors that significantly predicted beauty judgments: a factor capturing intensity variation, a factor capturing the spatial frequency information, a factor capturing the luminance, a factor capturing the color information (saturation and the degree of redness, mainly), and a factor capturing directionality, particularly diagonal orientations. We also identified two feature factors that significantly predicted roughness judgments: a luminance factor (the same factor that influenced the beauty judgments) and a uniformity factor.

Our results are in good agreement with previous studies investigating the relationships between features and aesthetics. Previous studies have highlighted relationships between beauty ratings and low spatial frequencies (Soen et al., 1987; Kawamoto and Soen, 1993; Schira, 2003) or between pleasure and color information (Valdez and Mehrabian, 1994). We have confirmed that these relationships are present, and have extended the findings to patterned stimuli, rather than only completely uniform stimuli. The relationships we found between color information and beauty ratings agree with those found by Valdez and Mehrabian (1994) in the sense that more saturated and more intense colors are rated as being more beautiful. A relationship between intensity variation and artistic merit has previously been reported by Juricevic et al. (2010), and our findings generalize this finding to beauty ratings; also Spehar and Taylor (2013) and Spehar et al. (2015) found results similar to those of Juricevic et al. (2010).

It is interesting to compare the features we found with the features that were found to predict liking ratings in Liu et al. (2015), who used similar stimuli and computed an almost identical set of features, but used a different method of feature selection, namely a so-called Laplacian score. Both studies found that saturation is an important feature. Other features are different but highly related. For example, Liu et al. found that both Tamura coarseness and Tamura contrast were important, while our study found that Tamura roughness was important. Since Tamura roughness is high when both Tamura coarseness and Tamura contrast are high, it can be seen that the outcomes are very similar. Similarly, both Liu et al. (2015) and we find that high contrast at diagonal orientations is associated with beauty and liking. The difference is that Liu et al. (2015) find this with features based on the gray-level co-occurrence matrices (GLCMs), while we found this with Gabor-features. Finally, both studies found that low spatial frequencies predict higher preference, again through different features. While Liu et al. find several low-frequency GLCM-features, we find instead several low-frequency Fourier features (c_energy2 and c_energy3) and low-frequency Gabor features (O*s1). Overall, the correspondences between these outcomes are high, and the small differences can presumably be attributed to minor differences in stimulus selection, analysis procedure, and maybe the slightly different judgments observers had to make.

Some of the features employed in our study were designed to capture the roughness of textures, as is apparent from their names: roughness and coarseness. Surprisingly, these features appeared to bear little relation to the roughness ratings. Features that emerged with much stronger relationships to the roughness ratings were the uniformity measures, called “angular second moment features” (based on the GLCM). These features should be regarded as reflecting the smoothness-roughness information more effectively than any of the other features that we computed. The other feature factor that emerged was average intensity. A lower average intensity was related to higher roughness ratings. One way to explain this finding is that weathered, rough surfaces tend to be of low intensity, whereas unscratched, shiny surfaces tend to reflect much light and are consequently rated as smooth.

### Limitations of the Current Study and Future Directions

This study was exploratory in nature. As such, confirmation of the relationships we have found is desirable, for example by selectively manipulating the features that we reported as important for beauty and roughness judgments. Moreover, our analyses were performed on average data, over groups of subjects. In reality, there is no such thing as an average observer (Güçlütürk et al., 2016), so it may be worthwhile to investigate inter-individual and cross-cultural differences. For example, Van Egmond et al. (2009) found that some people prefer rough surfaces, while others prefer smooth surfaces, which may result in orthogonality between the judgments when aggregating over participants, as we did. By performing cluster analysis on the raw judgments (Güçlütürk et al., 2016) and subsequently performing factor analysis on clusters of participants, different patterns of responses may be discovered, an approach we may pursue in the future.

Our exploratory study enabled us to examine the effects of many computed visual texture features simultaneously. This allowed us to find features that influence beauty and roughness ratings that have not been reported before. But a limitation of this exploratory approach is that we did not systematically vary certain features, while controlling for the effects of others. Hence, it is possible that the features that grouped together on our feature factors did this not because of an inherent relationship between the features, but because they tend to be grouped together in our stimulus set, possibly because certain features tend to co-occur in real life. An example of the latter is our feature factor with high loadings of saturation and redness. Clearly, stimuli can be designed to have unsaturated red, so that the effects of both features can be dissociated. Indeed, an earlier study found that saturation is the main factor influencing preference (Valdez and Mehrabian, 1994). Hence, it is possible that saturation rather than redness is the feature responsible for the relationship of our color factor to the beauty ratings.

Future research could address the role that context plays in determining preferences. Numerous factors could play a role, such as the cultural or professional background of the participants. Indeed, an initial report on socio-cultural influences on aesthetics ratings has appeared (Zhang et al., 2006), pointing to the malleability of the aesthetic response. The spatial context may also play a role. In our experiments, textures were always presented in isolation and surrounded by a gray background. In real life, textures occur in complex environments and are often part of objects. Results might be affected by the presence of such aspects.

Even though there is evidence that semantics provide a strong cue to preferences (Martindale et al., 1990), future research into the affective processing of photographs and paintings should take into account the idea that the texture features present in such material may exert a substantial influence on beauty ratings.

### Beauty is Still in the Eye and Brain of the Beholder

Philosophers such as Plato (2000) and Hutcheson (1973) debated whether beauty is objective and inherent in the objects around us, or whether we impose beauty on the objects around us. The systematic relationships between features and judgments could be taken as evidence for Plato’s point of view. However, we like to adhere to the notion that beauty is computed by our brains based on signals coming from our senses and is therefore imposed by us on the objects in our environment. How can these distinctive viewpoints be reconciled? Our study has revealed systematic affective responses across participants to certain visual features present in textures. We suggest that prior experience of a person with certain stimuli (e.g., during early childhood) may influence his or her affective responses to similar stimuli encountered later in life. Hence, we propose that the systematicity that we have uncovered is the result of common factors in human ontogeny. However, we cannot exclude additional influences from phylogeny.

## Conclusion

A two-dimensional judgment space sufficed to capture most of the variability in perceived texture qualities, which suggests that observers use a relatively limited set of internal scales on which to base various judgments, including aesthetic ones.

For both axes of this judgment space, represented by judgments of beauty and roughness, we found that a number of computational texture features correlate well with these judgments. This suggests that perceived texture qualities—including the aesthetic appreciation—are sufficiently universal to be predictable based on the computed feature content of textures.

## Author Contributions

RHAHJ: conception, programming experiment, data acquisition, analysis, interpretation of data, writing; KVH: programming experiment, data acquisition, analysis, interpretation of data; ST: feature computation, interpretation of data, writing; RR: analysis, interpretation of data, writing; BH: conception, interpretation of data, FWC: conception, programming experiment, interpretation of data, writing, funding acquisition.

## Conflict of Interest Statement

The authors declare that the research was conducted in the absence of any commercial or financial relationships that could be construed as a potential conflict of interest.
