# Supplementary material for: Aesthetics by Numbers: Links between Perceived Texture Qualities and Computed Visual Texture Properties
Source: Front Hum Neurosci. 2016 Jul 21;10:343. doi: 10.3389/fnhum.2016.00343 (PMC4954813; doi:10.3389/fnhum.2016.00343)
Supplement: Supplementary file 1 [file DataSheet_1.doc]

Supplementary information to the paper ‘Aesthetics by numbers: Deriving perceived texture qualities from computed visual texture properties’

## Authors and Affiliations

Richard H.A.H. Jacobs^1,2^, Koen V. Haak^1,3^, Stefan Thumfart^4,5^, Remco Renken^1^,

Brian Henson^6^, Frans W. Cornelissen^1^

1. Laboratory for Experimental Ophthalmology, University Medical Center Groningen, University of Groningen, Groningen, the Netherlands
2. Donders Institute for Brain, Cognition and Behaviour, Donders Center for Cognition, Radboud University Nijmegen, the Netherlands
3. Donders Institute for Brain, Cognition and Behaviour, Centre for Cognitive Neuroimaging, Radboud University Nijmegen, the Netherlands
4. Profactor GmbH, Steyr-Gleink, Austria
5. Research Unit for Medical-Informatics, RISC Software GmbH, Johannes Kepler University Linz, Linz, Austria
6. School of Mechanical Engineering, University of Leeds, Leeds, United Kingdom

**Corresponding author**: Richard Jacobs, richardjacobs01@hotmail.com

Contents:

I. Consistency in beauty ratings for textures

II. Selection of textures and adjectives

III. Description of features

**I. Consistency in beauty ratings for textures**

# Abstract

There are suggestions in the literature that texture affects aesthetics. Interested in texture’s effects on beauty judgments, we set out to determine the consistency of these judgments, within and between subjects. We find that consistent beauty ratings can be obtained, even between subjects, provided that textures are chosen that are sufficiently diverse in terms of initial beauty ratings.

# Introduction

There are sporadic suggestions in the literature that texture information, in particular spatial frequencies or fractal dimension present in stimuli, affects emotional and aesthetic judgments (Aks & Sprott, 1996; Kawamoto & Soen, 1993; Schira, 2003; Soen, Shimada, & Akita, 1987) and brain responses (Delplanque, N'Diaye, Scherer, & Grandjean, 2007; Holmes, Winston, & Eimer, 2005; Rolls et al., 2003; Vuilleumier, Armony, Driver, & Dolan, 2003). This suggests that people may show systematic preferences for textures, despite their apparent affective neutrality. In the tactile domain, a systematic relationship between smoothness and preference for textures has already been demonstrated (Ekman, Hosman, & Lindstroem, 1965). To investigate further, in the visual domain, we perform two experiments asking for repeated beauty judgments to textures. In the first, we ask people to judge textures twice, after which we select the extreme textures on an individual basis for a third judgment, to see if this selection results in stronger correlations between ratings. Encouraged by these findings, we proceed to a second experiment, in which we select extreme textures in terms of group average beauty ratings. This allows for a correlation between average beauty ratings, over participants, to textures. Participants return to judge these textures twice more, and high correlations are found.

# Method

In the first study, six male and eight female colleagues participated. They viewed 305 textures, taken from our database (available on request), one by one on a 30’’ Apple Cinema HD Display, and rated them for beauty by moving a slider along a horizontal bar (right side = beautiful), in a self-paced task, under instructions to respond based on their first impression. Textures were presented against a grey background, in which they gradually faded (see figure 1). Judgments took place on separate days.

In the second study, a different but overlapping set of 300 textures is drawn from the same large texture database. Twelve male and twelve female participants enrolled in exchange for course credits, and all but seven females returned for a second and third rating, which took place on a later day, and were separated by a number (about 9) of other judgments about the textures. The main difference with study 1 was that this time extreme textures were selected based on group average beauty ratings in the first round. The twenty most and least liked textures were taken, as well as twenty from the middle of the range.


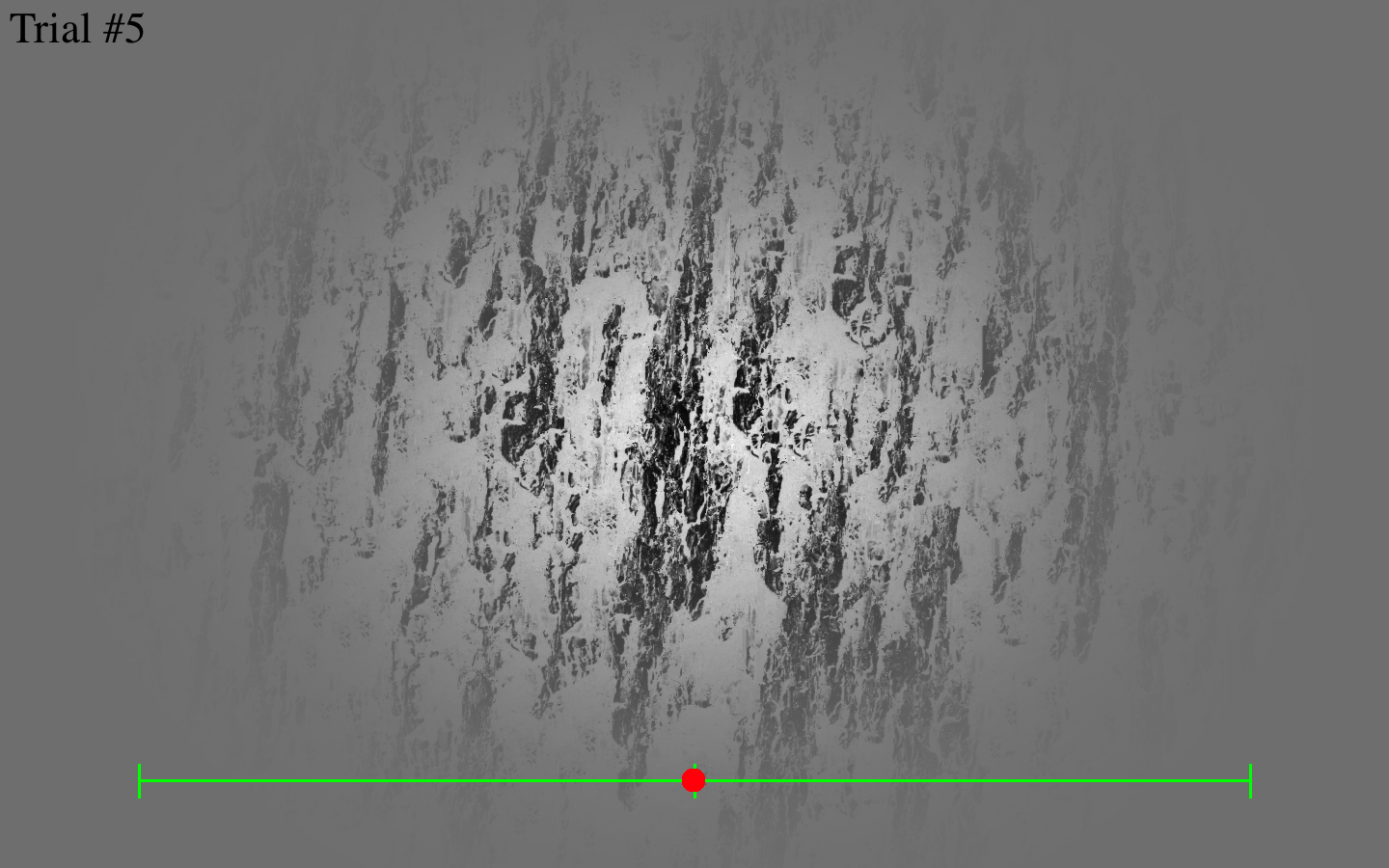


**Figure 1.** Example texture, as shown on screen, with a green slider bar at the bottom.

# Results


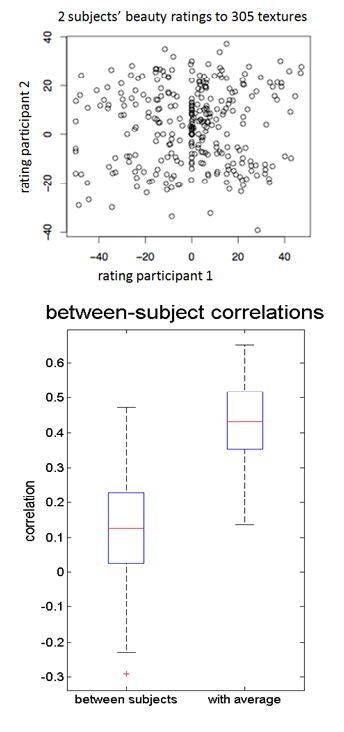


**Figure 2.** Representative example of two subjects’ ratings to 305 textures (top panel), showing low correlation. Bottom panel: average between-subjects correlation and spread, and the correlations with the average beauty rating, in the second session, study 1.


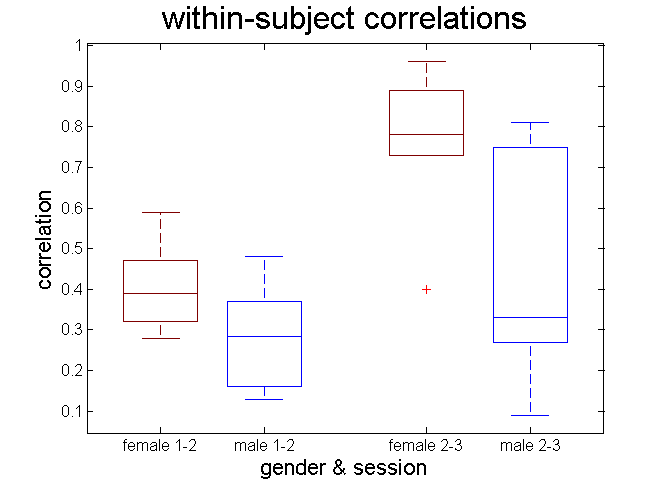


**Figure 3.** Within-subject correlations (r), calculated between the first and the second session (left) and the second and third session (right), for females (blue) and males (orange). Within-subject correlations are higher than the between-subject correlations above (figure 2, right panel). The selection of extremes for the third session appears to further enhance correlations.


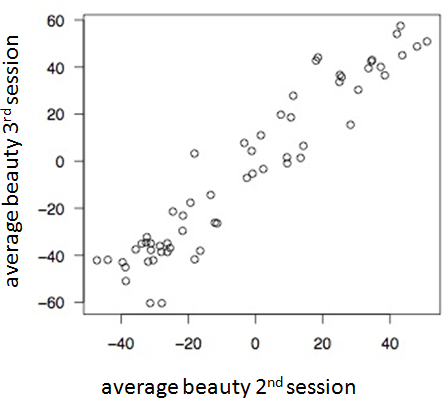


**Figure 4.** Correlation between mean ratings, over subjects, from the second and third session. An r-value of .95 was obtained.

Beauty ratings between different people are close to 0 (figure 2). Within subjects, correlations between repeated beauty ratings reach r-values of 0.3 (figure 3). After a pre-selection of the individually most and least liked textures, correlations between repeated beauty ratings reach values around 0.5 (figure 3). Pre-selecting based on group-averaged ratings results in a high correlation (r = 0.95) between the means of repeated ratings of textures, over subjects (figure 4).

# Discussion

When taking a random sample of textures, correlations between beauty ratings from different people are quite low (figure 2). Within subjects, correlations are better (r ≈ .5; figure 3, left part), but we aimed for higher reliability, because we want to obtain reliable beauty judgements in a brain imaging experiment, based on a pre-selection of textures. By selecting the extremes at an individual level, a strong enhancement of within-subject correlations can be obtained (figure 3, right part), showing that there is some consistency in the ratings of the textures, and beauty judgments are not merely a result of random fluctuations in, for example, the state of the viewer. Selecting extremes on beauty can even be done on group-average ratings, resulting in consistent average ratings (r = .95; figure 4) between sessions, over subjects.

There is a suggestion that females are more consistent in their beauty ratings than males. The difference is mostly due to two low-scoring males, however, so we think the issue of gender differences in consistency in (texture) beauty ratings, warrants further investigation.

In a similar study (Hofel & Jacobsen, 2003), subjects judged abstract graphic patterns multiple times over several days. Beauty judgments appeared to be quite inconsistent in that study, but consistency improved when judgments to these items were repeated. The authors interpreted this to be a reflection of subjects’ desire to maintain consistency, and thus not a genuine aesthetic response. Similar factors may have been at work here. Although mnemonic factors may play a role, we think the consistency in ratings to extremely rated textures is a valuable starting point for further research.

Vessel and Rubin (Vessel & Rubin, 2010) investigated beauty ratings of abstract and concrete pictures, and found that ratings were more reliable for the concrete pictures. This result was interpreted as indicative for an important role for semantics in aesthetic ratings. The pictures of objects used in these experiments appear far from random, however. It appears that when making photographs, the photographer already makes a pre-selection for the most and least beautiful pictures, and this may result in more extreme pictures being used for real objects than for abstract figures, which are often computer-generated. Our results suggest that such differences could result in more consistent beauty ratings to the real-world objects. The relevant factor may thus be extremity of the beauty value assigned to a stimulus, rather than semantics per se.

### II. Description of features

Literature suggests a large number of different methods for extracting features from textures. The performance of these methods depends on the application requirements.

In 1973, Haralick suggested to use **Gray Level Co-occurrence Matrix** (GLCM) based statistical measures for classification of textures (Haralick, 1979). The main assumption behind the idea of the GLCM is, to compute texture feature based on the gray level distribution of pixel pairs separated by a fixed displacement vector d. A GLCM entry at position (i, j) indicates how many times a pixel with gray level i is separated from a pixel of gray level j by the displacement vector d. The set of GLCMs computed for different displacement vectors are used to compute 14 statistical properties like Entropy, Energy, Homogeneity et cetera. The computation of texture properties based on GLCMs suffer from some problems. First, there is no automatic method for the appropriate selection of the displacement vector, even though this choice is highly important for the results. Second, the resulting features cannot be directly interpreted by a human observer, what violates the criteria of visual interpretability. Despite these drawbacks the set of GLCM based texture features has become one of the most widely used texture features and is still relevant for a large number of applications.


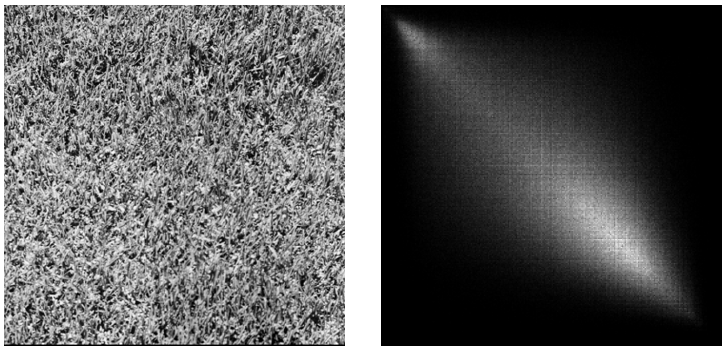


**Figure 5.** GLCM (right) of texture 99 from the Brodatz texture album (Brodatz & Textures, 1966)

In 1978, **Tamura** et al. tried to solve one major drawback of GLCM (see figure 5) based texture features by designing texture features that are directly visually interpretable (Tamura, Mori, & Yamawaki, 1978). They ended up with 6 texture features that were designed to match the results of a psychological experiment on texture classification. The features, namely coarseness, contrast, directionality, line-likeness, regularity and roughness have been widely used, especially in the field of Content Based Image Retrieval (CBIR). The computation of the Tamura features follows no general idea but is simply designed to receive perceptually interpretable quantitative texture measures.

Amadasun and King suggested the use of a **neighbourhood gray-tone difference matrix** (NGTDM) to compute texture measures correlated with human perception (Amadasun & King, 1989). Contrary to Tamura et al. their measures are all based on the NGTDM and not a selection of different concepts, which is useful regarding the computational performance. A NGTDM is a one dimensional matrix. The entry at index g stores the sum of gray-level differences of all pixels with gray-level g and their spatial neighbourhood. Based on this data the following features are computed: coarseness, contrast, busyness, complexity and texture strength. Amadasun and King did show the relevance of their features for human texture perception by comparing the measures to ranking experiments done by human subjects.

Considering that textures often consist of periodically placed texture elements the **autocorrelation function** (ACF) is an obvious choice for analysis (Haralick, 1979). The ACF is used to describe the periodicity of the texture by doing an element-wise multiplication of the image with a shifted version of itself. If the input texture is a periodic one, the resulting autocorrelation function can be used to extract features like regularity and coarseness. If the ACF is computed for a non-periodic texture, the result is dominated by a single peak. The width and elongation of the doming peak can be used to determine the coarseness and directionality of the texture. Figure 6 depicts the autocorrelation function for a periodic and non-periodic texture. Regarding our aim to compute at least perceptually relevant features, the publication of Fujii et al. (Fujii, Sugi, & Ando, 2003) is of particular interest. They propose a method of extracting the measures contrast, coarseness and regularity from the ACF of a texture and did prove strong correlation of the computed measures with the results of a psychological experiment.


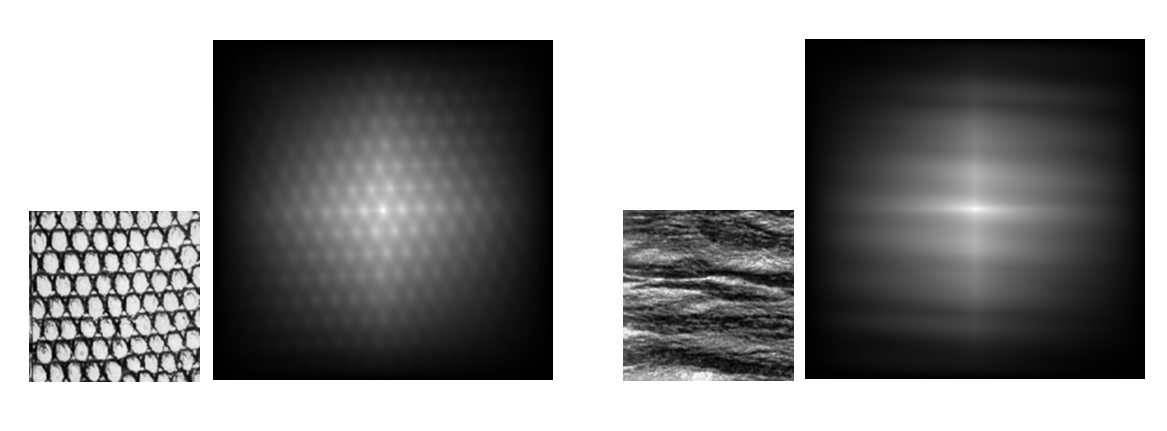


**Figure 6.**  ACF for a periodic (left) and non-periodic (right) texture samples from the Brodatz album (Brodatz & Textures, 1966)

The **power spectrum** of the **Fourier Transform** is closely related to the ACF (Haralick, 1979; Tuceryan & Jain, 1998)[[1]](#footnote-2). Consequently Fourier Domain based features are also used to compute texture measures like coarseness or directionality. This is done by computing the energy of circular bands around DC with different radius or by computing the energy of a wedge with its peaks placed at the DC Fourier coefficient (Tuceryan & Jain, 1998). The main disadvantage of the Fourier Transform is that spatial image information is lost. To reduce this effect, one can think of applying windowed Fourier transformation on parts of the texture image. Another possibility is to use spatially localized filters like the Gabor filter discussed later.


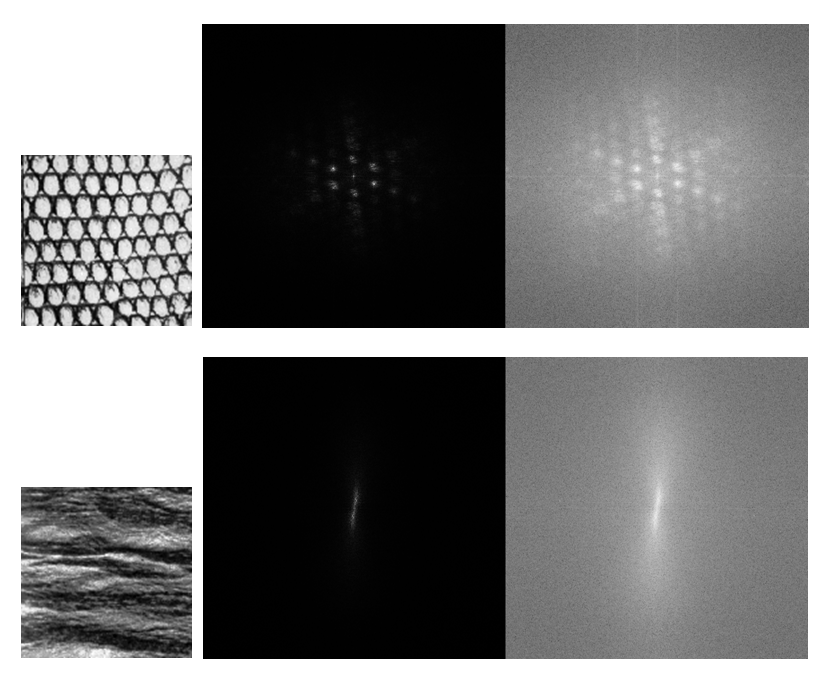


**Figure 7.**  Power spectrum (middle), logarithmic scaled Power spectrum (right) of Fourier transform for texture samples from the Brodatz album (left) (Brodatz & Textures, 1966).

If we take into account experiments on visual processing in the human brain, spatial domain filters are suggested to provide an adequate possibility for extracting texture information from visual images in a physiologically plausible way. There is a wide range of different filters and models to combine the filter responses in a more or less sophisticated way. An interesting comparison of different filter techniques with respect to texture classification was presented by Randen and Husoy (Randen & Husoy, 1999).

The filter masks presented by **Laws** (Laws, 1980) enable us to efficiently extract spatial texture information by convolving the image with different filter kernels. The filter masks are constructed by combining a horizontal and vertical one-dimensional filter:

$$\left(\begin{matrix} 1 \\ 4 \\ 6 \\ 4 \\ 1 \end{matrix}\right)\cdot\left(\begin{matrix} -1 & 0 & 2 & 0 & -1 \end{matrix}\right)=\left(\begin{matrix} -1 & 0 & 2 & 0 & -1 \\ -4 & 0 & 8 & 0 & -4 \\ -6 & 0 & 12 & 0 & -1 \\ -4 & 0 & 8 & 0 & -4 \\ -1 & 0 & 2 & 0 & -1 \end{matrix}\right)$$

h_1_ = [1, 4, 6, 4, 1]

h_2_ = [-1, -2, 0, 4, 1]

h_3_ = [-1, 0, 2, 0, -1]

h_4_ = [-1, 2, 0, -2, 1]

h_5_ = [1, -4, 6, -4, 1]

Based on this construction principle, the number of resulting filter masks is 25 for the 5 proposed separable one-dimensional filters. These 25 masks are applied to the texture image. Before the filter responses can finally be combined to the actual segmentation image, a local neighbourhood averaging is done.

Since Daugman found out that the receptive fields of cortical simple cells are best modelled using two-dimensional **Gabor filters** (Daugman, 1980, 1988), a lot of models, which try to simulate the human visual processing in the human brain, are based on Gabor filter banks (see figure 8).


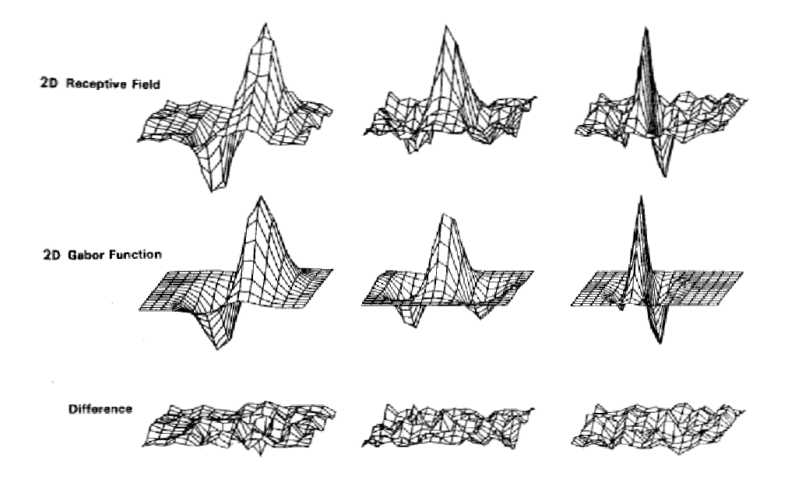


**Figure 8.**  The top row shows receptive field properties of simple cells in the cat visual cortex. The middle row contains the best fitting elementary Gabor filters. The bottom row displays the fitting error (Daugman, 1988).

A two-dimensional Gabor function consists of a sinusoidal plane wave of a certain frequency and orientation modulated by a Gaussian envelope. The Gabor function at an arbitrary orientation *θ_0_* can be obtained by a rigid rotation of the x-y plane by *θ_0_* (Tuceryan & Jain, 1998) (see figure 9).


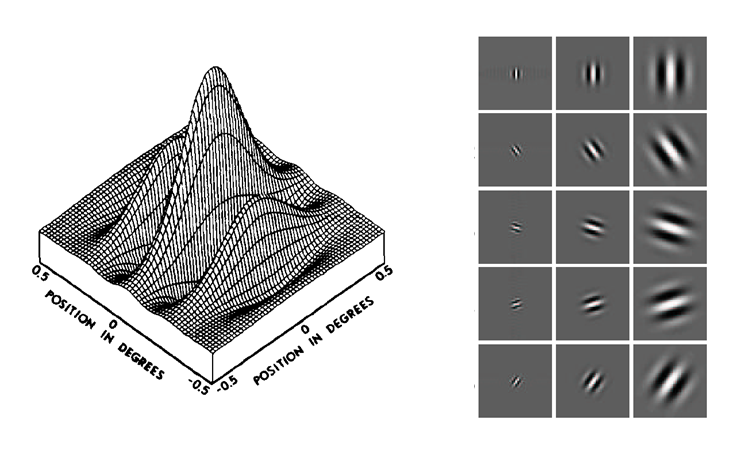


**Figure 9.**  A Gabor filter profile (left) and a Gabor filter bank (right) consisting of Gabor filters of different scales and orientations (Daugman, 1988).

The main difference between frequency analysis of the texture image using feature computation based on the Fourier power spectrum and using Gabor filters is that the Fourier coefficients depend on the entire image (global information) whereas Gabor filters can be used for local spatial frequency analysis.

If Gabor filters are used for image classification, segmentation or feature extraction in a first step a Gabor filter bank, consisting of Gabor filters of different scale and orientation (figure 9), is applied. The filter responses, also termed Gabor feature vectors, can be used directly as input for a segmentation or classification model (Bovik, Clark, & Geisler, 1990; Serre, Wolf, Bileschi, Riesenhuber, & Poggio, 2007). Usually, the raw Gabor feature vectors are processed and combined to more compact representations. For a comparison of texture features based on Gabor filters see (Grigorescu, Petkov, & Kruizinga, 2002).

### III. Judgment space with three retained components

In our paper we report judgment space with two retained components, since parallel analysis indicated that two components should be retained. However, the scree plots reveal that more than 15% of the variance in judgments is accounted for by a third component (in both semantic differential studies), so it may be worth looking at the solution with three components too. The plots of the the judgment loadings on the first and third components in the three-component solution are displayed in figure 10.
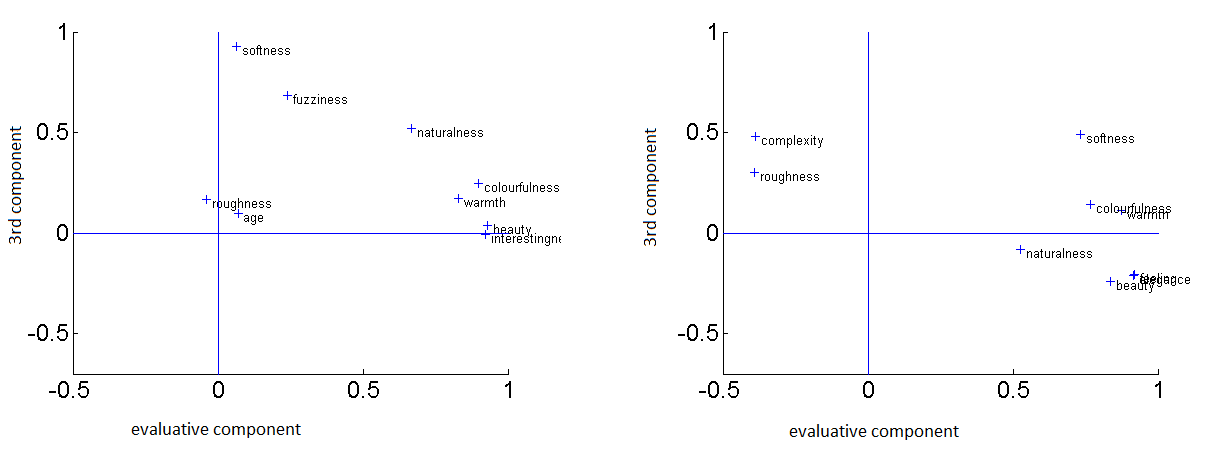


**Figure 10. Judgment loadings on the first and third components.** While softness and fuzziness appear to make up a nice third component in the first experiment (left panel), a matching solution is lacking in the second experiment (right panel), where all loadings on the third component appear quite low, and no judgments load exclusively on the third component.

To us, the lack of correspondence between the two experiments in the judgment loadings on the third component, and also the virtual absence of exclusive loadings on the third component in the second experiment, confirm that the third component does not contribute reliable information about judgment space, as already suggested by the parallel analysis.

# REFERENCES

Aks, D. J., & Sprott, J. C. (1996). Quantifying Aesthetic Preference for Chaotic Patterns. *Empirical studies of the arts, 14*, 1-6.

Amadasun, M., & King, R. (1989). Textural features corresponding to textural properties. *Systems, Man and Cybernetics, IEEE Transactions on, 19*(5), 1264-1274.

Bovik, A. C., Clark, M., & Geisler, W. S. (1990). Multichannel texture analysis using localized spatial filters. *IEEE Transactions on Pattern Analysis and Machine Intelligence, 12*(1), 55-73.

Brodatz, P., & Textures, A. (1966). A photographic album for artists and designers. *New York*.

Daugman, J. G. (1980). Two-dimensional spectral analysis of cortical receptive field profiles. *Vision Research, 20*(10), 847.

Daugman, J. G. (1988). Complete discrete 2-d Gabor transforms by neural networks for imageanalysis and compression. *IEEE Transactions on Acoustics, Speech and signal processing, 36*(7), 1169-1179.

Delplanque, S., N'Diaye, K., Scherer, K., & Grandjean, D. (2007). Spatial frequencies or emotional effects? A systematic measure of spatial frequencies for IAPS pictures by a discrete wavelet analysis. *Journal of Neuroscience Methods, 165*(1), 144-150.

Ekman, G., Hosman, J., & Lindstroem, B. (1965). Roughness, Smoothness, and Preference: a Study of Quantitative Relations in Individual Subjects. *J Exp Psychol, 70*, 18-26.

Fujii, K., Sugi, S., & Ando, Y. (2003). Textural properties corresponding to visual perception based on the correlation mechanism in the visual system. *Psychological Research, 67*(3), 197-208.

Grigorescu, S. E., Petkov, N., & Kruizinga, P. (2002). Comparison of texture features based on Gabor filters. *IEEE Transactions on Image processing, 11*(10), 1160-1167.

Haralick, R. M. (1979). Statistical and structural approaches to texture. *Proceedings of the IEEE, 67*(5), 786-804.

Hofel, L., & Jacobsen, T. (2003). Temporal stability and consistency of aesthetic judgments of beauty of formal graphic patterns. *Perceptual and Motor Skills, 96*(1), 30-32.

Holmes, A., Winston, J. S., & Eimer, M. (2005). The role of spatial frequency information for ERP components sensitive to faces and emotional facial expression. *Brain Research - Cognitive Brain Research, 25*(2), 508-520.

Kawamoto, N., & Soen, T. (1993). Objective evaluation of color design. *COLOR research and application, 18*, 260-266.

Laws, K. I. (1980). Rapid texture identification. *Image processing for missile guidance*, 376-380.

Randen, T., & Husoy, J. H. (1999). Filtering for texture classification: A comparative study. *IEEE Transactions on Pattern Analysis and Machine Intelligence, 21*(4), 291-310.

Rolls, E. T., O'Doherty, J., Kringelbach, M. L., Francis, S., Bowtell, R., & McGlone, F. (2003). Representations of pleasant and painful touch in the human orbitofrontal and cingulate cortices. *Cereb Cortex, 13*(3), 308-317.

Schira, G. (2003). Texture preference and global frequency magnitudes. *Environment and Planning B, Planning and Design, 30*, 297-318.

Serre, T., Wolf, L., Bileschi, S., Riesenhuber, M., & Poggio, T. (2007). Robust object recognition with cortex-like mechanisms. *IEEE Transactions on Pattern Analysis and Machine Intelligence, 29*(3), 411-426.

Soen, T., Shimada, T., & Akita, M. (1987). Objective evaluation of color design. *COLOR research and application, 12*(4), 187-195.

Tamura, H., Mori, S., & Yamawaki, T. (1978). Textural features corresponding to visual perception. *IEEE Transactions on Systems, Man and Cybernetics, 8*(6), 460-473.

Tuceryan, M., & Jain, A. (1998). Texture analysis. In C. Chen, L. Pau, & P. Wang (Eds.), *The Handbook of Pattern Recognition and Computer Vision* (2nd ed., pp. 207-248). New Jersey: World Scientific Publishing Co.

Vessel, E. A., & Rubin, N. (2010). Beauty and the beholder: Highly individual taste for abstract, but not real-world images. *Journal of Vision, 10*(2), 1-14.

Vuilleumier, P., Armony, J. L., Driver, J., & Dolan, R. J. (2003). Distinct spatial frequency sensitivities for processing faces and emotional expressions. *Nat Neurosci, 6*(6), 624-631.

1. “The relationship between the autocorrelation function and the power spectral density function is well known: they are Fourier transforms of one another”(Haralick, 1979) [↑](#footnote-ref-2)
